# A rare presentation of endogenous human chorionic gonadotrophin associated severe ovarian hyperstimulation in the second trimester: a case report

**DOI:** 10.1186/s13256-021-02936-w

**Published:** 2021-07-07

**Authors:** Abraham Fessehaye, Wondimu Gudu

**Affiliations:** Department of Obstetrics and Gynecology, Saint Paul’s Hospital Millennium Medical College, Addis Ababa, Ethiopia

**Keywords:** OHSS, Severe OHSS, Ovarian hyperstimulation syndrome

## Abstract

**Background:**

Ovarian hyperstimulation syndrome is usually an iatrogenic and potentially life-threatening disease. It develops following ovulation induction and use of *in vitro* fertilization techniques.

**Case summary:**

A 32-year-old primigravida Ethiopian woman presented at 15 weeks gestation with a history of progressive bilateral leg swelling and abdominal pain of 2 weeks duration. She had triplet pregnancy and conceived through *in vitro* fertilization. She was managed in intensive care unit.

**Conclusion:**

Patients with multiple pregnancy following *in vitro* fertilization conception can have ovarian hyperstimulation syndrome as late as 15 weeks gestation. Hence, frequent follow-up should be continued to detect early signs of OHSS to avoid further complications and need of intensive care unit care.

## Background

Ovarian hyperstimulation syndrome (OHSS) is usually an iatrogenic and potentially life-threatening disease, usually a complication during ovulation induction in *in vitro* fertilization (IVF) embryo transfer [1]. Spontaneous OHSS (sOHSS) has similar clinical presentations as OHSS and is a rare event [2]. The exact cause is unknown; however, vasoactive substances such as vascular endothelial growth factor, interleukins, endothelin-1, and tumor necrosis factor-α secreted by the ovaries may play a role in increased vascular permeability [3]. We report a rare case of severe OHSS in the second trimester.

## Case presentation

A 32-year-old primigravida Ethiopian woman presented at 15 weeks of gestation with a history of progressive bilateral leg swelling and abdominal pain that started 2 weeks back. A week after her initial presentation, she experienced cough and shortness of breath, which continued for a week. She had no history of weight loss. Her pregnancy was conceived by IVF, and it was a triplet pregnancy. She didn’t have similar complaint on early follow-up during and after IVF. She was risk stratified with meticulous evaluation after embryo transfer and had follow-up evaluations every 15 days. She had history of treatment for pulmonary tuberculosis (TB) a year back, and she was declared cured at that time. She had no history of cardiovascular disease, nor kidney disease.

At presentation, she had distended abdomen with bilateral lower abdominal tenderness. Pelvic ultrasound was done and revealed bilateral polycystic enlarged ovaries measuring right ovary 12 × 11 cm and left ovary 11 × 13 cm. The pregnancy was viable-fetal heart beat was positive for fetuses. She had also ascites up  to Morrison’s pouch. A conclusive diagnosis of OHSS was made, and she was admitted for conservative management. Chest X-ray was done and showed miliary-pattern infiltrates bilaterally. Her erythrocyte sedimentation rate (ESR), complete blood count (CBC) profile, liver function test, renal function test, and coagulation profile, all were normal. Few days after admission, her shortness of breath worsened. Upon physical examination, she was in  severe respiratory distress. Her oxygen saturation was 70%. She had tachycardia of 120 beats per minute and tachypnea of 50 breaths per minute. Her blood pressure was 120/100 mmHg.

With a revised diagnosis of severe OHSS with acute respiratory distress syndrome to rule out miliary tuberculosis, she was transfered to intensive care unit (ICU) and was put on mechanical ventilator. She was provided 80 mg of Lasix intravenous stat. She was provided crystalloids—3 Liters of normal saline. She was also started on anti-TB empirically, with additional diagnosis of disseminated TB. GeneXpert xpert test was negative. Bedside echocardiography was also done and was interpreted as normal. Thyroid function test was not updated. Later on, a diagnosis of severe hospital-acquired infection-sepsis was made as  patient's condition  deteriorated. After 2 weeks of stay in ICU, she sadly passed away—she succumbed to refractory septic shock due to the severe hospital aquired infection.

## Discussion

Ovarian hyperstimulation syndrome (OHSS) has two distinct types: early, occurring between 3 and 7 days after human chorionic gonadotrophin (hCG) ovulation trigger; and late, occurring between 12 and 17 days after hCG [4].OHSS is self-limiting, and complete resolution usually occurs in 10–14 days from the beginning of initial symptoms[5].

Early OHSS is a consequence of exogenous hCG administration before ovum pick-up and is usually related to overstimulation of the ovaries by gonadotrophins, whereas late OHSS is induced by endogenous hCG produced by a growing pregnancy; therefore, it only occurs in pregnant patients, especially those with multiple pregnancy [6]. Our case is had a late type that occurred in triplet IVF pregnancy at gestational age of 15 weeks.

Persistent second trimester OHSS is an unusual presentation, with only sparse cases reported in the literature [6]. Ujvari and colleagues reported a case of twin pregnancy conceived by IVF presenting with bloating, abdominal tenderness, and significant ascites warranting paracentesis at 15 weeks [7]. O’Brien *et al*. reported a case of late-onset OHSS discovered at 17 weeks in a fetus with trisomy 21 and terminated at 20 weeks by dilatation and curettage [8]. In 2017, Torky *et al*. reported an unusually prolonged course of ovarian hyperstimulation syndrome persisting until 22 weeks of gestation [9]. Recently, in 2019, Gui J *et al*. reported a case of spontaneous OHSS at gestational age of 17 weeks [2].

Conservative and symptomatic medical treatment is sufficient in the majority of patients, with intensive care admission in severe cases. Surgery is indicated only in extreme cases, such as ovarian torsion, internal hemorrhage, or in cases of a ruptured cyst, and should be done by an experienced surgeon because the hyperstimulated ovaries are very friable and vascular [10].

The definition of severe OHSS is defined according to revised criteria proposed by Golan and Navot [6]: abdominal pain, enlargement of ovary greater than 5 cm, and ascites (ultrasonographically diagnosed) or hydrothorax. In addition, one of the following criteria had to be met: hematocrit 45%, white blood cells 15,000/ml, oliguria (urinary output less than 500 ml/24 hours), elevated liver enzymes (above our laboratory normal values), dyspnea, anasarca, or acute renal failure [11]. Our patient fulfilled the criteria for severe OHSS—she had abdominal pain, enlarged and tender ovaries, ascites, dyspnea, and acute respiratory distress syndrome. However; OHSS should not be the “default diagnosis” for women presenting with abdominal pain during fertility treatment. A number of differentials, including pelvic infection, pelvic abscess, appendicitis, ovarian torsion or cyst rupture, bowel perforation, and ectopic pregnancy, should be also considered [12]. Ovarian cyst rupture with significant hemorrhage and ovarian torsion were a possible differentials in our case, but her hematocrit throughout was normal. The other ultrasound features of ovarian torsion, including absent flow on Doppler ultrasound and whirlpool features, were also absent. Acute exacerbation of tuberculosis was the other possible differential in our case, but GeneXpert was negative and the patient did not show improvement despite being put on anti-TB medication for 2 weeks.

## Conclusion

Endogenous hCG-associated persistent second-trimester OHSS is an unusual presentation. Patients with multiple pregnancy following IVF conception can have OHSS syndrome as late as 15 weeks gestation. Hence, frequent follow-up should be continued to detect early signs of OHSS and  avoid further complications and need of ICU care.

## Data Availability

All supporting documents are submitted along with the case report.
